# Maternal and foetal immune responses of cattle following an experimental challenge with *Neospora caninum* at day 70 of gestation

**DOI:** 10.1186/1297-9716-43-38

**Published:** 2012-04-26

**Authors:** Paul M Bartley, Stephen E Wright, Stephen W Maley, Colin N Macaldowie, Mintu Nath, Clare M Hamilton, Frank Katzer, David Buxton, Elisabeth A Innes

**Affiliations:** 1 Moredun Research Institute, Pentlands Science Park, Bush Loan, Midlothian, EH26 0PZ, Scotland, United Kingdom; 2 Biomathematics and Statistics Scotland, James Clerk Maxwell Building, The King’s Buildings, Edinburgh, EH9 3JZ, Scotland, United Kingdom; 3 Veterinary Sciences Centre, School of Veterinary Medicine, University College Dublin, Belfield, Dublin 4, Ireland

**Keywords:** *Neospora caninum*, Cattle, Early gestation, Maternal – foetal cellular immune

## Abstract

The immune responses of pregnant cattle and their foetuses were examined following inoculation on day 70 of gestation either intravenously (iv) (group 1) or subcutaneously (sc) (group 2) with live NC1 strain tachyzoites or with Vero cells (control) (group 3). Peripheral blood mononuclear cell (PBMC) responses to *Neospora* antigen and foetal viability were assessed throughout the experiment. Two animals from each group were sacrificed at 14, 28, 42 and 56 days post inoculation (pi). At post mortem, maternal lymph nodes, spleen and PBMC and when possible foetal spleen, thymus and PBMC samples were collected for analysis. Inoculation with NC1 (iv and sc) lead to foetal deaths in all group 1 dams (6/6) and in 3/6 group 2 dams from day 28pi; statistically significant (*p* ≤ 0.05) increases in cell-mediated immune (CMI) responses including antigen-specific cell proliferation and IFN-γ production as well as increased levels of IL-4, IL-10 and IL-12 were observed in challenged dams compared to the group 3 animals. Lymph node samples from the group 2 animals carrying live foetuses showed greater levels of cellular proliferation as well as significantly (*p* ≤ 0.05) higher levels of IFN-γ compared to the dams in group 2 carrying dead foetuses. Foetal spleen, thymus and PBMC samples demonstrated cellular proliferation as well as IFN-γ, IL-4, IL-10 and IL-12 production following mitogenic stimulation with Con A from day 14pi (day 84 gestation) onwards. This study shows that the generation of robust peripheral and local maternal CMI responses (lymphoproliferation, IFN-γ) may inhibit the vertical transmission of the parasite.

## Introduction

The protozoan parasite *Neospora caninum* is a major cause of abortion and reproductive failure in cattle worldwide. The most common route of infection with *N. caninum* appears to be the transplacental (vertical) transmission of the parasite from mother to foetus; this may result in abortion or the birth of clinically normal but persistently infected offspring [1,2]. Horizontal transmission of the parasite may occur in intermediate hosts through the ingestion of oocysts (shed by a definitive host i.e. dog) in contaminated feed and water [3], potentially leading to point source outbreaks (abortion storms) of neosporosis. Previous studies in cattle have shown that *N. caninum* infections can be maintained over several generations through vertical transmission of the parasite [1,4], Moen *et al*. (1998) demonstrated that as a result of a primary infection, cattle were 3–7 times more likely to abort than uninfected animals [5]. However, animals that have aborted due to neosporosis are less likely to abort due to the parasite during subsequent pregnancies, compared to cows undergoing *N. caninum* infection during their first pregnancy [6], suggesting that a certain level of protective immunity builds following infection. Experimental data by Innes *et al*., (2001) [7] demonstrated that exposure of cattle to *Neospora* prior to pregnancy protected against the vertical transmission of the parasite following an experimental challenge with *N. caninum* during pregnancy. Other factors influencing the outcome of *N. caninum* infections in pregnant cattle include; the quantity and duration of the parasitaemia [8], the parasite strain (as some have been shown to be more virulent than others, in cattle) [9], the immune status of the dam and the gestational age of the foetus at the time of infection [7,8]. Experimental infections of pregnant cattle have shown that foetal death may occur when dams were challenged with *N. caninum* tachyzoites at day 70 of gestation [10,11], while a challenge administered around mid gestation resulted in the vertical transmission of the parasite, but no foetal death [12,13]. These observations would suggest that the timing of a parasitaemia during pregnancy is critical in the clinical outcome and will likely be influenced by both the maternal and foetal immune responses to the parasite. Work carried out by Williams *et al*. (2000); Collantes-Fernandez *et al*., (2006) and Rosbottom *et al*., (2007) [10,14,15] would support this conclusion.

The intracellular nature of *N. caninum* suggests that a cell-mediated immune (CMI) response is likely to be important to protect the host [12]. Increasing experimental data from pregnant cattle has confirmed this [7,15-18]. Work by Bartley *et al*., (2004) [13] demonstrated a strong CMI response in dams and foetuses challenged with *N. caninum* on day 140 of gestation. Although, no foetal deaths were recorded, vertical transmission of the parasite occurred and the maternal and foetal immune responses appeared to contribute to the resolution of infection. Numerous other studies have illustrated the importance of a pro-inflammatory T-helper (Th)-1 type response, interferon-γ (IFN-γ) in particular has been shown to be crucial in controlling infection both *in vivo*[15,16,19] and *in vitro*[20-22]. The timing and location of these pro-inflammatory immune responses has also been shown to be critical to the clinical outcome of a *Neospora* infection in cattle. Work by Maley *et al*., (2006) [23] demonstrated in cattle experimentally challenged with *Neospora* on day 70 of gestation; that the infiltration of large numbers of immune cells and increased levels of expression of IFN-γ mRNA in the placenta lead to foetal death and abortion.

In this study, we compared the maternal and foetal immune responses in cattle inoculated either intravenously (iv) or subcutaneous (sc), with live *N. caninum* (NC1 strain) tachyzoites at day 70 of gestation. A serial examination of the maternal and foetal immune responses was conducted looking at *Neospora* specific cell proliferation and cytokine production in PBMC and lymph node samples following experimental challenge.

## Materials and methods

### Animals, inoculum and experimental design

Twenty four pregnant Holstein-Friesian cattle aged 1.3 to 4 years and seronegative for *N. caninum*, *Toxoplasma gondii*, bovine viral diarrhoea virus, infectious bovine rhinotracheitis and *Leptospira hardjo* were assigned into three groups. Pregnancy and foetal viability was confirmed in all experimental animals by ultrasound scanning 36 days after insemination. On day 70 of gestation, group 1 dams (n = 8) received an intravenous (iv) inoculation in the right jugular vein of 5×10^8^ live *N. caninum* (NC1 isolate) tachyzoites. Group 2 dams (n = 8) received a subcutaneous (sc) inoculation of 5×10^8^ live *N. caninum* (NC1 isolate) tachyzoites over the left pre-femoral lymph node. Group 3 (n = 8), the control animals each received an iv inoculation of 5×10^6^ Vero cells. This dose of Vero cells was used, as it was the equivalent number of cells present in the parasite inocula. Blood was collected by weekly jugular venipuncture throughout the experiment for immunological analysis. Two animals from each group were sacrificed at days 14, 28, 42 and 56 post inoculation (pi). At post mortem samples of left pre-femoral lymph node (LPF), right pre-femoral lymph node (RPF), left uterine lymph node (LUL), right uterine lymph node (RUL), mesenteric lymph node (MLN), retropharyngeal lymph node (RLN), spleen and peripheral blood mononuclear cells (PBMC) were collected from each dam; When possible spleen, thymus and PBMC samples were collected from the foetuses.

### Preparation of cells for immunological assays

Single cell suspensions of PBMC were prepared as previously described [7]. Samples from lymph nodes collected at post mortem were prepared as previously described [13]. In brief, excess fat was trimmed from the tissues, which were then cut into small pieces and placed in 10 ml wash buffer (Hank’s balanced salt solution (HBSS) supplemented with 2% foetal bovine serum (FBS) (Labtech International, Ringmer, UK) 100 IU/ml penicillin and 50μg/ml streptomycin) (Northumbria Biologicals, Cramlington, UK), placed in a stomacher bag (Seward Medical, Northampton, UK) and homogenised for 10 seconds. The resultant cell suspension was decanted into a sterile universal through sterile lens tissue to remove clumps of cells, washed twice by repeated centrifugation at 260 × *g*, counted using a Neubauer haemocytometer and resuspended at a final concentration of 2×10^6^ cells/ml in cell culture media (CCM) (Iscoves modified Dulbecco’s media (IMDM) (Gibco, Paisley, UK) supplemented with 10% FBS, 100 IU/ml penicillin and 50μg/ml streptomycin).

### Cell proliferation assays

Single cell suspensions of both PBMC and lymph node tissues were treated as previously described [13]. In brief, equal volumes (100 μl) of cells (2×10^6^/ml) and antigen were added in quadruplicate to 96-well round bottom plates (Nunc, Roskilde, Denmark). Water-soluble *N. caninum* tachyzoite antigen (NCA) [7] was used at a final protein concentration of 1μg/ml, the T-cell mitogen concanavalin A (Con A) was used as a positive control at a final concentration of 5μg/ml, CCM alone was used as a negative control to determine the background level of proliferation. A Vero cell lysate antigen at 1 μg/ml was used as a control antigen. The cultures were incubated at 37°C in a humidified 5% CO_2_ atmosphere for 5 days. The cultures were pulsed with 18.5 kBq ^3^ H Thymidine/well (Amersham Biosciences, Little Chalfont, UK) for the final 18 hours, before being harvested onto glass-fibre filters (Canberra Packard, Meriden, CT, USA) and the cell-associated radioactivity was quantified using a MATRIX 96^TM^ gas proportional counter.

### Cytokine responses

Duplicate cell proliferation assays were prepared to those described above. Cell free supernatants were collected after 4 days incubation, to measure the levels of secreted cytokines (interferon-gamma (IFN-γ), interleukin-4 (IL-4), IL-10 and IL-12). The supernatant samples were stored at −20°C prior to analysis.

### IFN-γ

Levels of IFN-γ production were quantified using a commercially available enzyme linked immunosorbent assay (ELISA) kit (CSL Veterinary, Parkville, Australia). A standard curve was generated using doubling dilutions of known quantities (ng/ml) of recombinant bovine IFN-γ (rBoIFN-γ) (Pfizer Animal Health, Parkville, Australia). Mean optical density (OD) values were plotted against ng/ml rBoIFN-γ and a standard regression curve was fitted to the data. Experimental samples were extrapolated against the standard regression curve to determine the levels of IFN-γ in the test samples.

### IL-10

Levels of bovine IL-10 were quantified using an ELISA method as previously described [24]. In brief, 96-well ELISA plates (Greiner, Stonehouse, UK) were coated with 50μl (4μg/ml) per well with a primary anti-bovine IL-10 capture antibody and incubated at room temperature overnight. The plates were washed x 5 using phosphate buffered saline (PBS) supplemented with 0.05% Tween 20 (PBS-T) between each step, with the exception of the final TMB – H_2_SO_4_ stage. The plates were blocked at room temperature for 1 hour with PBS-T supplemented with 3% bovine serum albumin (BSA). Samples and standards (50μl each) were added and incubated at room temperature for 1 hour. Plates were then coated with (1μg/ml) secondary biotinylated anti IL-10 antibody (Diluted in PBS-T 1%BSA) (50μl per well) and incubated at room temperature for 1 hour. Streptavidin-horseradish peroxidase (HRP) (Dako Cytomation, Glostrup, Denmark) diluted 1:500 in PBS-T 1%BSA (50μl/well) was added and incubated at room temperature for 45 minutes. Colour was developed by the addition of TMB (3,3′,5,5′-tetramethylbenzidine) substrate (Insight Biotech. Ltd., Wembley, UK) (100μl/well) and incubated in the dark for 10–15 minutes. Reactions were stopped by adding 50μl/well 1 M H_2_SO_4_. The plates were read at 450/650 nm using a MRX II plate reader (Dynex, East Grinstead, UK). Doubling dilutions of known quantities of recombinant bovine IL-10 (rBoIL-10) were used to generate a standard regression curve against which the test sample concentrations were extrapolated.

### IL-12

Interleukin-12 (IL-12) was quantified using the same method described above for the detection of IL-10, with the following changes. A primary anti IL-12 capture antibody (4μg/ml) was used along with a secondary biotinylated anti IL-12 antibody (8μg/ml). Known quantities of recombinant ovine IL-12 (rOvIL-12) were used as standards and standard regression curve was fitted to the data [25].

### IL-4

Interleukin-4 (IL-4) was quantified using the same method described above for the detection of IL-10 and IL-12, with the following changes. A primary anti IL-4 capture antibody (6μg/ml) was used along with a secondary biotinylated anti IL-4 antibody (2μg/ml). Known quantities of recombinant bovine IL-4 (rBoIL-4) were used as standards and a standard regression curve was fitted to the data [26].

All primary and secondary antibodies used for the capture and detection of IL-4, IL-10 and IL-12 were purchased from AbD Serotec, (Oxford, UK). All rBoIL-4, rBoIL-10 and rOvIL-12 cytokines (Moredun Research Institute, Edinburgh, UK)

### Foetal serology

At post mortem examination blood was drawn from the foetuses (when available) into non-heparinised evacuated tubes (Vacutainer, Becton Dickinson, Oxford,UK) and allowed to clot before centrifugation at 2000 *x****g*** for 15 minutes, the serum was removed and stored at −20°C prior to being tested for IgM and IgG to *N. caninum* by an indirect fluorescent antibody test (IFAT), as previously described [27]; an IFAT titre of ≥1:64 was considered positive.

### Statistical analysis

The maternal cell proliferation data (PBMC, lymph nodes and spleen) and IFN-γ ELISA data were analysed with a linear mixed model, using a first-order autoregressive model to specify the temporal covariance structure. Both the proliferation and IFN-γ ELISA data were normalised by logarithmic transformation (base 10) prior to the analysis. The linear mixed model included the animal effect as a random effect and the treatment, day and the interaction effect of treatment and day as fixed effects. Parameters of the linear mixed models were estimated using the REML method and *p*-values were estimated using the modified *F*-statistic. If the *F*-statistic was statistically significant (*p* ≤ 0.05), two-sided probabilities for each treatment comparison were obtained; these probabilities were then adjusted using a False Discovery Rate approach [28]. This value, denoted in this paper as *p*_*f*_, therefore summarises the strength of evidence for there being a real difference in a way analogous to a standard *p*-value.

The foetal proliferation and IFN-γ data (PBMC and spleen) were analysed using non-parametric Kruskal-Wallis one way analysis of variance (ANOVA) using treatment as a grouping factor. No data was available for group 1 for the foetal thymus, (proliferation and IFN-γ ELISA) hence a two sample non-parametric Mann–Whitney test was conducted. All statistical analyses were carried out using GenStat 13^th^ Edition software (VSN International, Hemel Hempstead, UK)

## Results

### Clinical observations, pathology and foetal mortality

Foetal viability following experimental challenge is shown in Additional file 1. In group 1 (iv) viable foetuses were only seen on day 14 pi, both foetuses were found dead on day 28 pi and no foetuses were found on days 42 and 56 pi. In group 2 (sc) two viable foetuses were found on day 14 pi and one live and one dead foetus on days 28, 42 and 56 pi. In the group 3 (control) animals, two live foetuses were found at each time point. The maternal serology and histopathology data from this experiment is described in Macaldowie *et al*., (2004) *Neospora caninum* parasites were only demonstrated in placental and foetal tissues from challenged animals carrying dead foetuses and they were associated with lesions [11].

### Maternal PBMC

#### Cell proliferation

The results from the PBMC proliferation assays from group 1 (iv) demonstrated statistically significantly higher mean levels of antigen-specific proliferation on days 14 (*p*_*f*_ = 0.020), 21 (*p*_*f*_ = 0.004), 42 (*p*_*f*_ = 0.043) and 56 pi (*p*_*f*_ < 0.001) compared to group 3 (control). The mean cell proliferation results from group 2 (sc) were found to be statistically significantly higher than group 3 on days 7 (*p*_*f*_ = 0.004), 14 (*p*_*f*_ = 0.011), 21 (*p*_*f*_ < 0.001), 42 (*p*_*f*_ < 0.001), 49 (*p*_*f*_ = 0.020) and 56 pi (*p*_*f*_ < 0.001). A comparison of the data from group 1 and group 2 showed that the mean antigen-specific proliferation in group 2 was higher than group 1 on day 7 pi (*p*_*f*_ = 0.003). At subsequent time points, the mean level of antigen specific PBMC proliferation of group 2 animals were higher in comparison to group 1 animals, though these differences were not statistically significant. ( Additional file 2).

### Cytokine responses

The levels of antigen-specific cytokine production was determined using the cell-free supernatants from maternal PBMC, lymph nodes and spleen samples following stimulation with NCA for 4 days

### IFN-γ

The log_10_ transformed antigen specific-IFN-γ data from PBMC cultured with NCA ( Additional file 3). Group 1 (iv) showed a rise in IFN-γ from days 7 to 21 pi and reached a peak on day 28 pi, then declined to baseline levels on day 56 pi. Though group 1 maintained an elevated level of IFN-γ compared to the group 3 (control) animals, there was no evidence of a difference between the mean IFN- γ levels of the two groups (*p*_*f*_ = 0.107). In group 2 (sc), levels of IFN-γ were consistently higher than other groups at all time points and reached a peak on day 21 pi. The mean level of IFN-γ from group 2 was statistically significantly different from that of group 3 (*p*_*f*_ = 0.005), though there was no evidence of a difference between the mean IFN-γ levels of group 1 and 2 (*p*_*f*_ = 0.118).

### IL-4

In group 1 (iv) demonstrable antigen-specific IL-4 was observed in PBMC samples of one dam on day 14 pi (1.543U/ml), IL-4 production was seen in PBMC from both group 1 dams on day 28 pi (8.37U/ml and 2.54U/ml); while on days 42 and 56 pi PBMC samples from group 1 were below the detection level of the ELISA. Detectable levels of IL-4 were only seen in the PBMC from one of the group 2 (sc) animals on day 28 pi (carrying a dead foetus), these levels were much lower (0.518U/ml) than those seen in group 1, though no statistical differences were observed between groups 1 and 2. The levels of IL-4 in the group 3 (control) PBMC were either below the detection threshold of the ELISA at 0.114U/ml or only just detectable (data not shown).

### IL-10

Demonstrable antigen-specific IL-10 was observed in PBMC from one group 2 (sc) animal carrying a dead foetus on 42 pi (0.286U/ml). All PBMC samples from groups 1 (iv) and 3 (control) were below the detection threshold (0.17U/ml) of the ELISA at all time points (data not shown).

### IL-12

Levels of antigen-specific IL-12 were demonstrable in most PBMC samples collected from all three groups on days 14, 28 and 42 pi. Group 2 (sc) animals demonstrated higher levels of IL-12 than the other two groups. On day 28 pi the group 2 dam carrying the dead foetus demonstrated 39.1U/ml while, 94.3U/ml was recorded from the dam carrying the live foetus. By day 56 pi levels of IL-12 were either below the detection threshold (0.3U/ml) or only just detectable in all three groups (data not shown).

### Maternal lymph nodes and spleen

#### Cell proliferation

The log_10_ transformed antigen specific proliferation in maternal lymph nodes and spleen samples following stimulation with NCA. On day 14 pi, samples of LPF from group 2 (sc) had significantly (*p*_*f*_ = 0.010) higher mean proliferation than group 3 (control). While mean proliferation from spleen samples from groups 1 (iv) and group 2 were significantly higher (*p* = 0.05 and *p* = 0.047 respectively) than group 3 ( Additional file 4).

On day 28 pi, samples of LPF from group 1 and 2 had significantly higher mean proliferation than group 3 (*p*_*f*_ = 0.006 and *p*_*f*_ = 0.025 respectively), while samples of RLN and RPF from group 1 showed higher values than groups 2 and 3 (*p*_*f*_ = 0.023 and *p* = 0.05 respectively) and (*p* = 0.033 and *p* = 0.015 respectively) ( Additional file 4).

On day 42 pi, samples of LPF from group 2 and 3 had significantly higher mean proliferation than the group 1 (*p*_*f*_ = 0.005 and *p*_*f*_ = 0.031 respectively) ( Additional file 4).

On day 56 pi, group 2 demonstrated statistically significantly higher mean proliferation than group 3 from RPF (*p*_*f*_ = 0.051), LPF (*p*_*f*_ < 0.001), RLN (*p*_*f*_ = 0.023) and RUL (*p* = 0.021) and MLN (*p* = 0.007) samples. Group 2 also demonstrated significantly higher mean proliferation than group 1 for LPF samples (*p*_*f*_ <0.001), while group 1 had significantly higher mean proliferation than group 3 for RLN (*p*_*f*_ = 0.023) and RUL (*p* = 0.021) samples ( Additional file 4).

### Cytokine responses

#### IFN-γ

Following the stimulation of cells from spleen and lymph nodes with NCA for 4 days; group 1 (iv) and group 2 (sc) both showed detectable levels of antigen specific-IFN-γ (ng/ml) in all samples tested from day 14 pi onwards, while group 3 (control) produced almost undetectable levels of IFN-γ at each of the time points tested ( Additional file 5). The mean levels of IFN-γ from group 1 was statistically significantly higher than group 3 for RPF (*p*_*f*_ = 0.001), RUL (*p*_*f*_ < 0.001), LUL (*p*_*f*_ = 0.013), RLN (*p*_*f*_ = 0.003) and spleen (*p*_*f*_ = 0.001). Similarly, the mean levels of IFN-γ from group 2 were statistically significantly higher than group 3 for RPF (*p*_*f*_ = 0.004), LPF (*p*_*f*_ = 0.005), RUL (*p*_*f*_ = 0.005), RLN (*p*_*f*_ = 0.009) and spleen (*p*_*f*_ = 0.004). There was no evidence in the present data that the mean IFN-γ values of group 1 and 2 were different for any of the lymph node or spleen.

#### IL-4

Group 1 (iv) showed demonstrable antigen-specific IL-4 from both dams for all spleen and lymph node samples (except for LPF and RPF – only one dam responded) on day 28 pi and in LUL of one dam on 42 pi. All samples tested from group 2 (sc) and group 3 (control) were either below the detection threshold of the ELISA at 0.114U/ml or only just detectable and hence no statistical analysis was undertaken (data not shown).

#### IL-10

Group 1 (iv) was the only group with demonstrable antigen-specific IL-10, this was seen in LUL (2.61U/ml) and RLN (1.27U/ml) samples on day 42 pi, All samples tested from group 2 (sc) and group 3 (control) were below the detection threshold of the ELISA and hence no statistical analysis was undertaken (data not shown).

#### IL-12

Group 1 (iv) had demonstrable levels of antigen-specific IL-12 in RPF, LUL, RUL, MLN, RLN and spleen on days 14, 28 and 42 pi, with the highest levels being observed on day 42pi (126.75U/ml, 210.55U/ml, 98.00U/ml, 114.71U/ml, 307.9U/ml and 137.09U/ml respectively). By day 56 pi the levels of IL-12 had dropped below the detection threshold of the ELISA (0.3U/ml). Group 2 (sc) demonstrated levels of IL-12 below or barely above the threshold in most samples on days 14 and 28 pi. Levels of IL-12 peaked on day 42 pi, in the RLN and spleen, (23.64U/ml and 33.23U/ml respectively); by day 56 pi levels of IL-12 had dropped below threshold. Group 3 (control) showed low but detectable levels of antigen specific IL-12 from samples at all time points. Due to paucity of data, no statistical analysis was undertaken (data not shown).

### Foetal PBMC, thymus and spleen

#### Cell proliferation

Samples collected from the foetuses for immunological analysis are shown in Table 1. No *Neospora* antigen-specific cell proliferation was demonstrated from any of the samples from any of the foetuses tested.

**Table 1:** Foetal samples collected at post mortem examination

	Number of foetal samples collected/Total number of foetuses									Number of foetal samples collected/Total number of foetuses
	Group 1 (iv)			Group 1 (iv)	Group 2 (sc)			Group 2 (sc)	Group 3 (Control)			Group 3 (Control)
Day pi	PBMC	Thymus	Spleen	PBMC	Thymus	Spleen	PBMC	Thymus	Spleen
14	2/2	0/2	2/2	2/2	0/2	2/2	2/2	0/2	2/2
28	0/2‡	0/2‡	0/2‡	0/2	1/2‡	1/2‡	0/2	2/2	2/2
42	0/2†	0/2†	0/2†	1/2‡	1/2‡	1/2‡	2/2	2/2	2/2
56	0/2†	0/2†	0/2†	1/2‡	1/2‡	1/2‡	0/2	2/2	2/2

‡ − Foetus found dead *in utero* (No Samples able to be collected). † − No foetus found (due to resorption or abortion).

**Figure 1:**
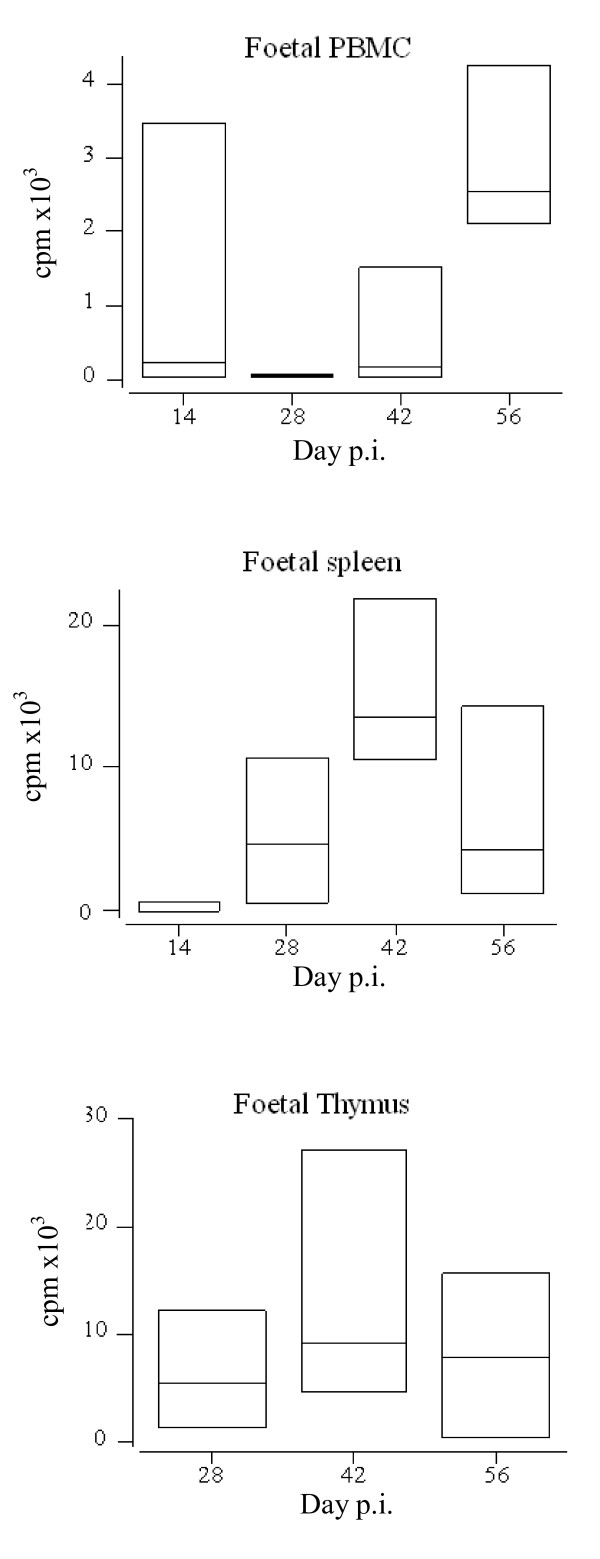
**Proliferative responses of foetal PBMC, spleen and thymus samples following stimulation with Con A for 5 days.** Samples of PBMC, spleen and thymus were collected at post mortem examination when available. Samples were stimulated with Con A for 5 days (37°C in a humidified 5% CO_2_ atmosphere), with 18.5 kBq ^3^ H Thymidine/well being added for the final 18 hours, before being harvested onto glass-fibre filters. The box plots illustrate the combined counts per minute (cpm) ×10^3^ values from the foetal samples from all three group s at each time point. The boxes represent the upper and lower values obtained for each sample, the horizontal line = the median value.

From day 14 pi (day 84 of gestation) PBMC samples showed proliferation following stimulation with Con A (Figure 1). On day 28 pi (day 98 of gestation) mitogenic proliferation was demonstrated in the spleen and thymus of foetuses from both group 2 (sc) and group 3 (control). On day 42 pi (day 112 of gestation) proliferative responses to Con A were continued to be seen in PBMC, thymus and spleen samples from the foetuses from both group 2 and group 3. On day 56 pi (day 126 of gestation) PBMC, spleen and thymus all showed proliferation following Con A stimulation (Figure 1). When the responses of the foetal tissues were compared, no statistical differences were observed between the groups in the levels of mitogenic proliferation.

### Cytokine responses

Following stimulation with NCA, levels of antigen-specific IFN-γ IL-4, IL-10 and IL-12 were below detectable limits in all the foetal samples tested.

### IFN-γ

The levels of IFN-γ following Con A stimulation on Day 14 pi (day 84 of gestation) were below detectable limits for all the samples tested. On days 28, 42 and 56 pi (days 98, 112 and 126 of gestation respectively) PBMC, spleen and thymus samples produced demonstrable quantities of IFN-γ, following stimulation with Con A. There were no significant differences observed in the levels of IFN-γ produced in Con A stimulated cells from any of the groups, at any of the time points tested (data not shown).

### IL-4

Following Con A stimulation, on day 14 pi all samples tested from all three groups were below the detection threshold of the ELISA. On day 28 pi (day 98 of gestation), production of IL-4 was demonstrated in one group 3 (control) foetal thymus sample (0.117U/ml). On day 42 pi (day 112 of gestation) one group 3 foetal thymus (0.178U/ml) and both group 3 spleen (0.221U/ml, 0.214U/ml) samples produced demonstrable IL-4. On day 56 pi (day 126 of gestation), IL-4 was produced by the thymus samples of all three foetuses (group 2 and 3) and in the PBMC samples from both group 3 foetuses (data not shown).

### IL-10

Following Con A stimulation, on day 14 pi all samples tested from all three groups were below the detection threshold of the ELISA. On day 28 pi (day 98 of gestation), IL-10 was produced by one group 3 (control) foetal spleen sample (0.587U/ml). On day 42 pi (day 112 of gestation) IL-10 was produced by group 2 (sc) and group 3 thymus samples (0.201U/ml and 0.194U/ml respectively); and in thymus, (0.359U/ml) spleen (0.947U/ml) and PBMC (1.681U/ml) samples from both group 2 and group 3 on day 56 pi (day 126 of gestation) (data not shown).

### IL-12

Following Con A stimulation, IL-12 was expressed in foetal spleen and PBMC samples from all three groups from as early as day 14 pi (day 84 of gestation). Production of IL-12 continued to be demonstrated in all tissues tested from both group 2 (sc) and group 3 (control); peaking in the thymus (8.66U/ml) and spleen (12.17U/ml) on day 28 pi (day 98 of gestation) and in PBMC (12.92U/ml) on day 56 pi (day 126 of gestation) (data not shown).

### Foetal serology

The results from the foetal anti-*Neospora* IgG IFAT show that on day 28 pi (day 98 of gestation) a serum sample collected from one of the foetuses from group 1 (iv) which was found dead *in utero*, gave a positive IFAT result with a titre of 1:128. On days 42 and 56 pi (days 112 and 126 of gestation respectively) no foetuses were present for sample collection. In groups 2 (sc) and 3 (control), serum samples were collected from live foetuses, these all tested negative for IgG IFAT (titre ≤1:64). All the samples tested from all the groups were negative (titre ≤1:64) for anti-*Neospora* IgM (data not shown).

### Comparison of maternal immune responses in group 2 (sc) dams carrying live or dead foetuses

**Figure 2:**
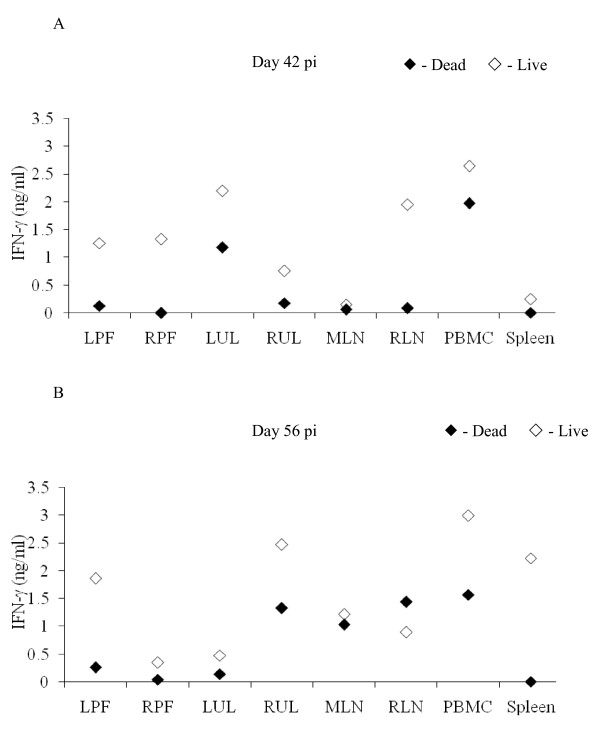
**Comparison of IFN-γ production by group 2 (sc) dams carrying live or dead foetuses.** Maternal lymph node, spleen and PBMC samples were collected at post mortem examination. Following stimulation with NCA for 4 days (37°C in a humidified 5% CO_2_ atmosphere), ELISA were performed to determine the concentration of IFN-γ produced. The data is expressed as ng/ml. –– Live, –– Dead.

The immune responses of the dams in group 2 (sc) carrying live or dead foetuses were compared; The dam carrying a live foetus demonstrated considerably higher levels of cellular proliferation in LUL and RUL on days 28, 42 and 56 pi compared to the dam carrying the dead foetus; while on days 42 and 56 pi higher levels of proliferation were also seen in the PBMC of the dams carrying the live foetus compared to the dams carrying the dead foetuses. Though there were no significant differences seen in the levels of maternal proliferation, when the log_10_ data were compared using a linear mixed model, the dams carrying the live foetuses generally demonstrated higher levels of cell proliferation in PBMC and lymph nodes stimulated with NCA, compared to the dams carrying the dead foetuses (data not shown). Comparisons were also made in the levels of antigen specific-cytokine responses (IFN-γ, IL-4, IL-10 and IL-12) in PBMC and lymph nodes of dams carrying live foetuses compared to the dams carrying dead foetuses in group 2. On day 28 pi, greater levels of antigen specific-IFN-γ were seen in samples from the LPF, LUL, RUL and RPF from dams carrying live foetuses compared to the dams carrying dead foetuses (data not shown). On days 42 (Figure 2A) and 56 pi, (Figure 2B) dams carrying live foetuses demonstrated increased levels of IFN-γ in all samples except RLN (on day 56 pi) compared to the dams carrying dead foetuses. When IFN-γ data from days 28, 42 and 56 pi were combined and analysed using a linear mixed model, the IFN-γ responses in the RPF and spleen of dams carrying live foetuses were statistically significantly higher than in dams carrying dead foetuses (*p* = 0.008 and *p* = 0.005 respectively). Dams carrying live foetuses also showed higher levels of antigen specific-IL-12 on day 28 pi (RPF, LUL, RUL, spleen and PBMC). On day 42 pi, the dam with the live foetus had higher levels of antigen specific-IL-12 in RLN (23.64U/ml), spleen (33.27U/ml) and PBMC (94.30U/ml) compared to the dam carrying a dead foetus (1.23U/ml, 13.9U/ml, and 39.11U/ml respectively). None of the dams carrying live foetuses had demonstrable antigen specific-IL-4 at any time point, while one dam carrying a dead foetus on day 42 pi showed antigen-specific IL-4 production in LUL, RUL, MLN, spleen and PBMC. No differences were seen in levels of antigen-specific IL-10 production between the dams carrying live and dead foetuses at any of the time points tested.

## Discussion

This paper examines the maternal and foetal cell-mediated immune (CMI) responses of pregnant cattle experimentally inoculated with live *N. caninum* tachyzoites on day 70 of gestation (for a full description of the clinical observations and pathological findings see Macaldowie *et al*., (2004) and Maley *et al*., (2006) respectively) [11,23]. The route of inoculation of *N. caninum* tachyzoites played an important role in the outcome of infection, with 100% of group 1 (iv) animals having dead, reabsorbed or aborted foetuses from day 28 pi, compared to only 50% of the group 2 (sc) animals. The dams in group 2 (sc) proved to be very interesting, allowing us to compare the CMI responses in dams carrying live compared to dead foetuses following an identical challenge. Although the number of animals in the experiment was limited these results will aid our understanding of a protective maternal immune response, which is critical for foetal survival, due to the immaturity of the foetal immune response at this stage of gestation. Mitogenic responses were detected in the foetuses from day 84 of gestation (day 14 pi) and anti-*Neospora* IgG was demonstrable from day 98 of gestation (day 28 pi).

### Maternal immune response

The CMI responses observed in the dams in this study provides further evidence that cell-mediated immunity and in particular IFN-γ is important in protection against the rapidly multiplying intracellular tachyzoite stage of the parasite. Our data would suggest that a strong Th1 type cell-mediated immune response in both the peripheral and uterine lymph nodes as well as in PBMC, in particular in the group 2 (sc) dams carrying live foetuses, appears to have been sufficient to prevent the transplacental transmission of the parasite. When the immune responses of the dams in group 2 (sc) carrying live and dead foetuses were compared, it was seen that dams carrying live foetuses tended to exhibit stronger antigen-specific lymphocyte proliferation, significantly higher levels of IFN-γ as well as increased IL-12 production in samples of PBMC and lymph nodes, compared to dams in the same group (sc) carrying dead foetuses. Work carried out by Williams *et al*., (2000) [10] demonstrated that infection of pregnant cattle at 10 weeks gestation with *N. caninum* was accompanied by high levels of IFN-γ and lymphoproliferative responses indicating a profound Th1 helper T-cell like response in PBMC. Rosbottom *et al*., (2007) [15] observed proliferation of CD4^+^ T-cells and the expression of IFN-γ and IL-4 in PBMC stimulated with *N. caninum* antigen in pregnant cattle challenged on day 70 of gestation.

However, when a strong immunological response occurs in the placenta during early gestation, this can be detrimental to the pregnancy. Davies *et al*., (2004) [29] demonstrated that the activation of the maternal mucosal immunity within the uterus during early placentome development, may lead to immune mediated abortion. During our study, the group 2 (sc) animals carrying dead foetuses (examined by Maley *et al*. (2006) [23]) demonstrated infiltration of large numbers of T lymphocytes, γδ T-cells, NK cells and IFN-γ mRNA, leading to extensive immune mediated damage (necrosis) of the placenta, compared to the dams carrying the live foetuses who showed mild or no infiltration of the immune cells, no demonstrable IFN-γ mRNA and consequently little or no immune mediated placental damage. Similar observations were made by Rosbottom *et al*., (2008) [30] who found in pregnant cattle, following a challenge with *N. caninum* in early gestation, foetal death was associated with extensive placental necrosis, which corresponded to CD4^+^ T-cell and macrophage infiltration and increased expression of IFN-γ.

### Route of infection

Our data shows that the route of inoculation plays a critical role in the outcome of infection, with iv inoculation (5x10^8^ live tachyzoites) leading to a greater incidence of foetal mortality than a sc inoculation with the same dose of parasites. This may be due in part to the haemotogenous nature of the iv inoculation, allowing the rapid transportation of the parasites to the placenta, before an effective maternal CMI response can be initiated to limit the spread of the tachyzoites. Comparing the immunological responses generated by the different routes of inoculation, group 2 animals (sc) demonstrated stronger proliferative responses in PBMC (by day 7 pi) and many of the lymph nodes (by day 14 pi) than the group 1 (iv) animals. Superior priming of the immune responses in the group 2 (sc) animals appears to have prevented the transplacental transmission of the parasite in 50% of cases. The animals in group 2 (sc) carrying live foetuses may have initiated an innate immune response more rapidly than the group 1 (iv) animals. This initiation is likely through the interaction of pathogen associated molecular patterns (PAMP) with toll like receptors (TLR) on the surface of antigen presenting cells (APC); this interaction induces a signalling pathway that will in turn lead to the activation of APC’s and the production of pro-inflammatory cytokines, as well as leading to the maturation and migration of dendritic cells (DC) to local draining lymph nodes [31]. This APC migration may have been more rapid in the group 2 (sc) animals, as they were inoculated over a lymph node (left pre femoral lymph node). To date there is little experimental evidence demonstrating a role for the innate immune response in bovine neosporosis; work by Boysen *et al*., (2006) [20] and Klevar *et al*., (2007) [32] demonstrated a role for Natural killer (NK) cells during early infection in calves, in producing IFN-γ, and observed NK cytotoxicity in *N. caninum* infected fibroblast cells. Work in mice by Dion *et al*., (2011) [33] demonstrated that both DC and macrophages secrete IL-12 following exposure to viable *N. caninum* parasites, and that interactions between T lymphocytes and DC were involved in inducing IFN-γ production by T lymphocytes. Evidence from the closely related parasite *Toxoplasma gondii* has demonstrated an important role for innate mechanisms in a protective immune response including TLR [34-36]. Our experiment demonstrates through serial analysis; differences in the PBMC as well as lymph node responses of animals experimentally challenged by two different routes of inoculation (iv and sc). These differences appear to have been sufficient to protect against the vertical transmission of the parasite in some sc inoculated animals, compared to the iv challenged animals where vertical transmission and foetal death occurred in all animals from day 28 pi onwards.

### Foetal immunology

As ruminants have a syndesmochorial placentation, which does not usually allow the transplacental passage of maternal immune factors including immunoglobulins and cytokines [37], any immune responses detected in the foetus are likely to be due to a response to an active infection *in utero*. Our study showed that foetuses at day 98 of gestation (day 28 pi) are capable of mounting a humoral (IgG) immune response against *Neospora*. This finding would agree with work by Senogles *et al*., (1979) [38] who demonstrated that at 3 months (approx 90 days) gestation bovine foetal PBMC contained B cells that labelled positively for the presence of IgG. By day 98 gestation, components of a cell-mediated immune response are also starting to develop. Our study demonstrated the presence of lymphoproliferative responses as well as the production of IFN-γ, IL-4, IL-10, and IL-12 being observed in foetal spleen, thymus and PBMC samples following mitogenic (Con A) stimulation. Schultz *et al*., (1973) [39] demonstrated in cattle the development of follicles of lymphoid thymus from as early as day 42 of gestation, while the spleen appears to develop from day 55 of gestation; while, Senogles *et al*., (1979) [38] demonstrated the presence of T-cells in the bovine foetal thymus, spleen and PBMC from 3 months of gestation. Work carried out by Hein *et al*., (1988) [40] showed that by day 120 of gestation bovine foetal lymphocytes were well developed and capable of mitogenic stimulation as well as the production of IL-2. Our work would suggest that this development occurs by around day 100 of gestation. The lack of any *Neospora*-specific CMI or humoral responses in the live foetuses from the group 2 (sc) dams was due to the fact that the foetuses were naïve to *Neospora*, due to no vertical transmission having occurred. The lack of histopathological lesions or demonstrable *Neospora* antigen in the placentomes or foetal tissues [11] indicates that vertical transmission had not occurred in the group 2 (sc) dams carrying live foetuses.

Other studies looking at the development of foetal immunology in cattle experimentally infected with *Neospora* have demonstrated that foetuses from infected dams at day 131 of gestation are capable of producing both Th1 and Th2 type cytokines [13,16]. Bartley *et al*. (2004) [9] showed that the foetuses from dams inoculated with *N. caninum* at mid gestation (day 140) are capable of mounting a specific cell-mediated immune response from 14 days post inoculation (day 154 gestation). While Andrianarivo *et al*. (2001) [17] demonstrated strong CMI responses 9 weeks post challenge in bovine foetuses (days 219–231 of gestation).

### Timing of infection

We demonstrate that experimental inoculation of cattle at day 70 of gestation can lead to foetal mortality between 14–28 days post inoculation (pi) (days 84–98 of gestation). Studies by Barr *et al*., (1994), Williams *et al*., (2000), Collantes-Fernandez *et al*. (2006) and Rosbottom *et al*., (2007, 2008) [14,30,41,42] have demonstrated that experimental infections with *N. caninum* in the first trimester of pregnancy leads to increased foetal mortality and higher parasite burdens and greater dissemination of parasites in foetal tissues than infections at mid or late pregnancy in cattle. Though Rojo-Montejo *et al*., 2009b observed no demonstrable foetopathy in cattle challenged on day 70 of gestation with the *Neospora* isolate Nc Spain 1 H [43]. While, Williams *et al*., (2000), Maley *et al*. (2003), Rosbottom *et al*., (2007) and Almeria *et al*., (2011) [10,12,15,44] demonstrated that a primary challenge in naïve pregnant heifers around mid gestation led to the transplacental transmission of the parasite but no foetal death. The results from these studies demonstrate that the timing of a primary infection during pregnancy is critical to the survival of the foetus. As gestation progresses the severity of a foetal infection decreases but the chances of congenital infection increases.

## Conclusions

Our study demonstrates the development of maternal and foetal immune responses in lymph nodes and PBMC following challenge with live *N. caninum* tachyzoites. The innate immune response in the group 2 (sc) dams carrying live foetuses appears to have lead to superior priming of a cell mediated immune response, which inhibited the vertical transmission of the parasite, compared to the group 2 dams carrying dead foetuses. The route of inoculation of *N. caninum* tachyzoites has an impact on the clinical outcome of pregnancy in cattle, with an iv route of inoculation resulting in greater incidence of foetal mortality and less efficient immune priming compared to a sc inoculation of the same number of parasites. Foetuses from day 84 of gestation are capable of producing cellular responses to the mitogen Con A; these include lymphocyte proliferation as well as cytokine (IFN-γ, IL-4, IL-10, and IL-12) production. Our data has also shown that by day 98 of gestation foetuses are able to mount a humoral (IgG) response to *Neospora*. However should vertical transmission of the parasite occur, the foetuses are still too immunologically immature to mount a protective immune response, resulting in foetal mortality.

## Abbreviations

iv, Intravenously; sc, Subcutaneously; PBMC, Peripheral blood mononuclear cell; CMI, Cell-mediated immune; IFN-γ, Interferon-gamma; IL-4, Interleukin-4; IL-10, Interleukin-10; IL-12, Interleukin-12; Con A, Concanavalin A; Th1, T-helper 1; Th2, T-helper 2; LPF, Left pre-femoral lymph node; RPF, Right pre-femoral lymph node; LUL, Left uterine lymph node; RUL, Right uterine lymph node; MLN, Mesenteric lymph node; RLN, Retropharyngeal lymph node; FBS, Foetal bovine serum; CCM, Cell culture media; IMDM, Iscoves modified Dulbecco’s media; NCA, N. caninum tachyzoite antigen; CO2, Carbon dioxide; ELISA, Enzyme linked immunosorbent assay; PBS, Phosphate buffered saline; BSA, Bovine serum albumin; TMB, 3,3′,5,5′-tetramethylbenzidine; H2SO4, Sulphuric acid; HRP, Horseradish peroxidase; IFAT, Indirect fluorescent antibody test; IgM, Immunoglobulin M; IgG, Immunoglobulin G; ANOVA, Analysis of variance.

## Competing interests

The authors declare that they have no competing interests.

## Authors’ contributions

PMB, SEW, SWM, CNM, DB and EAI made substantial contributions to the conception and design. PMB, CMH and EAI were involved in the acquisition of data. PMB and MN were involved in the analysis of the data. PMB, MN, FK and EAI have been involved in the drafting and critical review of the manuscript. All authors read and approved the final manuscript

## Supplementary Material

Additional file 1 Foetal viability results following either iv (group 1) or sc (group 2) inoculation with live NC1 strain tachyzoites. Click here for file

Additional file 2 Log_10_ transformed maternal PBMC proliferation data following stimulation with NCA for 5 days. Click here for file

Addition file 3 Log_10_ Transformed IFN-γ results from maternal PBMC following stimulation with NCA for 4 days. Click here for file

Additional file 4 Log_10_ transformed proliferative responses from maternal lymph nodes and spleen samples following stimulation with NCA for 5 days. Click here for file

Additional file 5 Levels of antigen specific-IFN-γ (ng/ml) produced by maternal lymph node and spleen samples following stimulation for 4 days with NCA. Click here for file
