# Supplementary material for: Maternal and foetal immune responses of cattle following an experimental challenge with Neospora caninum at day 70 of gestation
Source: Vet Res. 2012 Apr 26;43(1):38. doi: 10.1186/1297-9716-43-38 (PMC3416710; doi:10.1186/1297-9716-43-38)
Supplement: Additional file 1 — Foetal viability results following either iv (group 1) or sc (group 2) inoculation with live NC1 strain tachyzoites. [file 1297-9716-43-38-S1.doc]

Additional file 1: Foetal viability results following either iv (group 1) or sc (group 2) inoculation with live NC1 strain tachyzoites.

|  | | Days post inoculation  Number of live viable foetuses / Total number of foetuses | | | |
| --- | --- | --- | --- | --- | --- |
| Group | Inoculation | 14 | 28 | 42 | 56 |
| 1 (*n*= 8) | 5 × 10^8^ NC1 (iv) | 2 / 2 | 0 / 2‡ | 0 / 2† | 0 / 2† |
| 2 (*n*= 8) | 5 × 10^8^ NC1 (sc) | 2 / 2 | 1 / 2‡ | 1 / 2‡ | 1 / 2‡ |
| 3 (*n* = 8) | 5 × 10^6^ Vero cells | 2 / 2 | 2 / 2 | 2 / 2 | 2 / 2 |

‡ - Foetuses found dead in utero † - No foetus found (due to resorption or abortion)
