# Supplementary material for: Maternal and foetal immune responses of cattle following an experimental challenge with Neospora caninum at day 70 of gestation
Source: Vet Res. 2012 Apr 26;43(1):38. doi: 10.1186/1297-9716-43-38 (PMC3416710; doi:10.1186/1297-9716-43-38)
Supplement: Additional file 2 — Log10 transformed maternal PBMC proliferation data following stimulation with NCA for 5 days. [file 1297-9716-43-38-S2.doc]

Additional File 2: Log_10_ transformed maternal PBMC proliferation data following stimulation with NCA for 5 days.


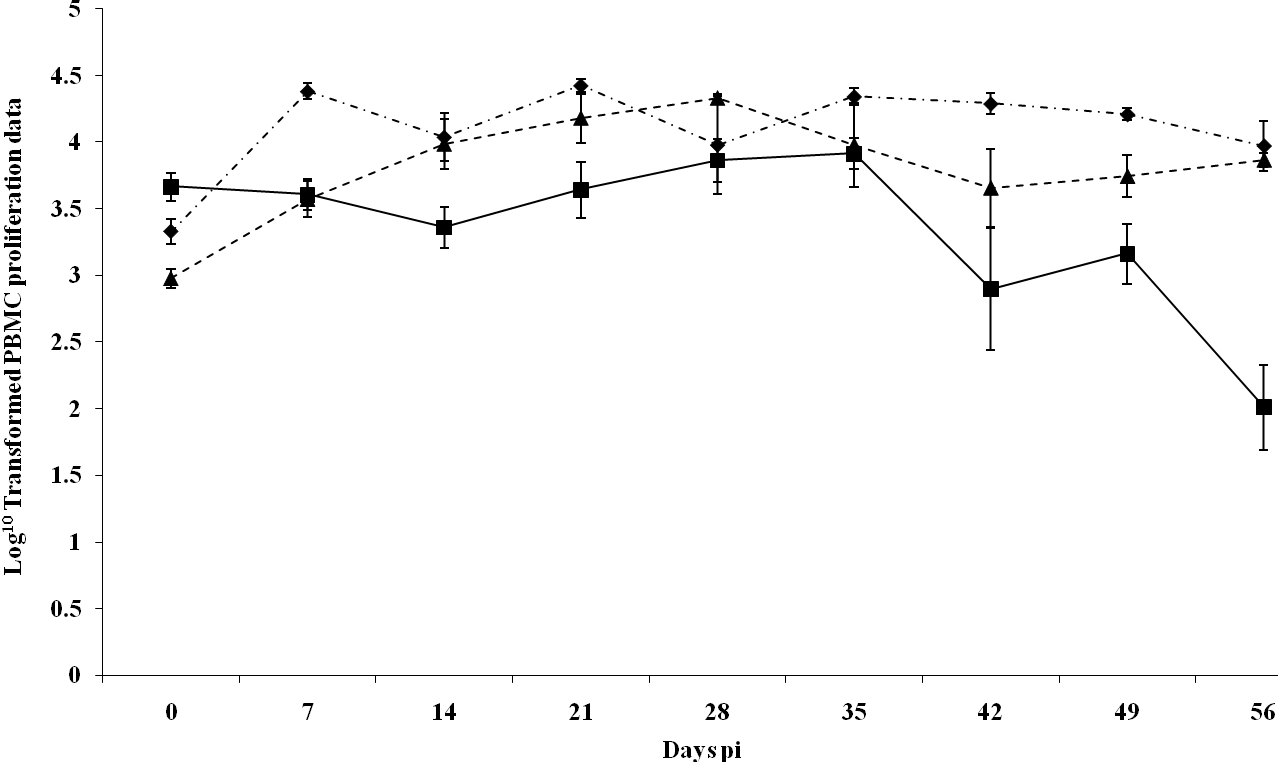


**Log_10_ transformed maternal PBMC proliferation data following stimulation with NCA for 5 days.**

Blood was collected by weekly venapuncture throughout the experiment and processed to determine levels of *Neospora*-specific proliferation. The processed cells were stimulated with NCA for 5 days (37 °C in a humidified 5% CO_2_ atmosphere), with 18.5kBq ^3^H Thymidine / well being added for the final 18 h, before being harvested onto glass-fibre filters. The data was then log^10^ transformed before analysis using a linear mixed model.

-▲- Group 1 (iv), -♦- Group 2 (sc), -■- Group 3 (Control)

Error Bars Error bars (± standard error of the mean (S.E.M.))
