# Supplementary material for: Maternal and foetal immune responses of cattle following an experimental challenge with Neospora caninum at day 70 of gestation
Source: Vet Res. 2012 Apr 26;43(1):38. doi: 10.1186/1297-9716-43-38 (PMC3416710; doi:10.1186/1297-9716-43-38)
Supplement: Addition file 3 — Log10 Transformed IFN-γ results from maternal PBMC following stimulation with NCA for 4 days. [file 1297-9716-43-38-S3.doc]

Additional file 3: Log_10_ Transformed IFN-γ results from maternal PBMC following stimulation with NCA for 4 days.


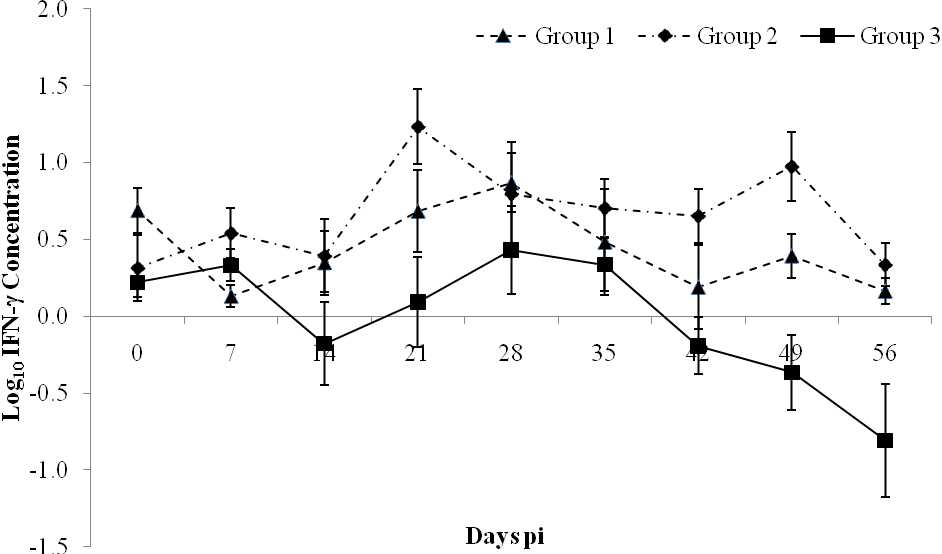


Blood was taken weekly to prepare PBMC. Following stimulation of PBMC with NCA for 4 days (37 °C in a humidified 5% CO_2_ atmosphere), ELISA were performed to determine the concentration of IFN-γ produced. The data was then log_10_ transformed before analysis using a linear mixed model. -▲- Group 1 (iv), -♦- Group 2 (sc), -■- Group 3 (Control). Error bars (± standard error (S. E.))
