# Supplementary material for: Maternal and foetal immune responses of cattle following an experimental challenge with Neospora caninum at day 70 of gestation
Source: Vet Res. 2012 Apr 26;43(1):38. doi: 10.1186/1297-9716-43-38 (PMC3416710; doi:10.1186/1297-9716-43-38)
Supplement: Additional file 4 — Log10 transformed proliferative responses from maternal lymph nodes and spleen samples following stimulation with NCA for 5 days. [file 1297-9716-43-38-S4.doc]

Additional File 4: Log_10_ transformed proliferative responses from maternal lymph nodes and spleen samples following stimulation with NCA for 5 days.

**A**  Day 14 pi


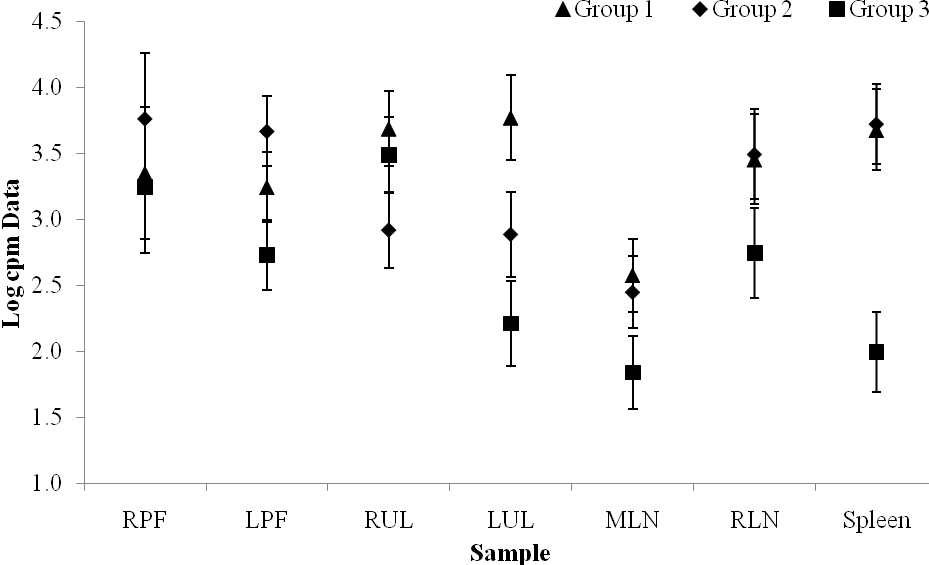


**B** Day 28 pi


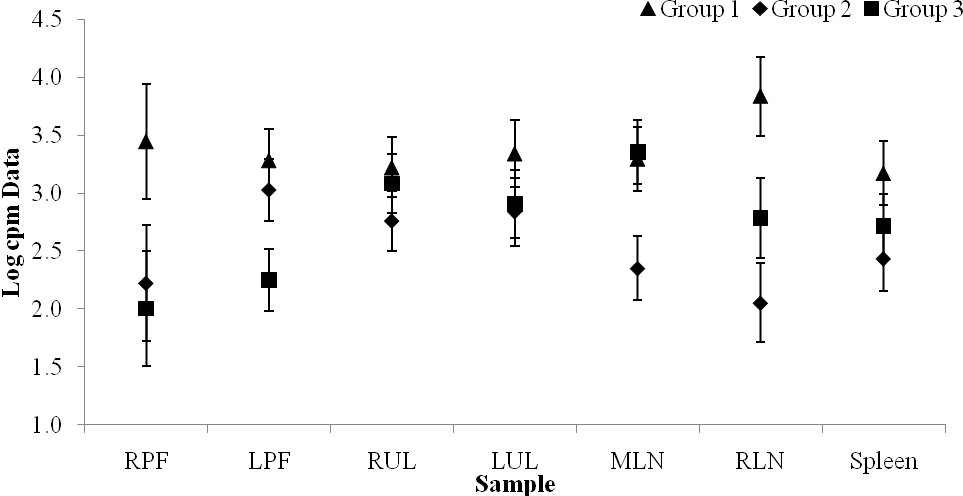


**C**  Day 42 pi


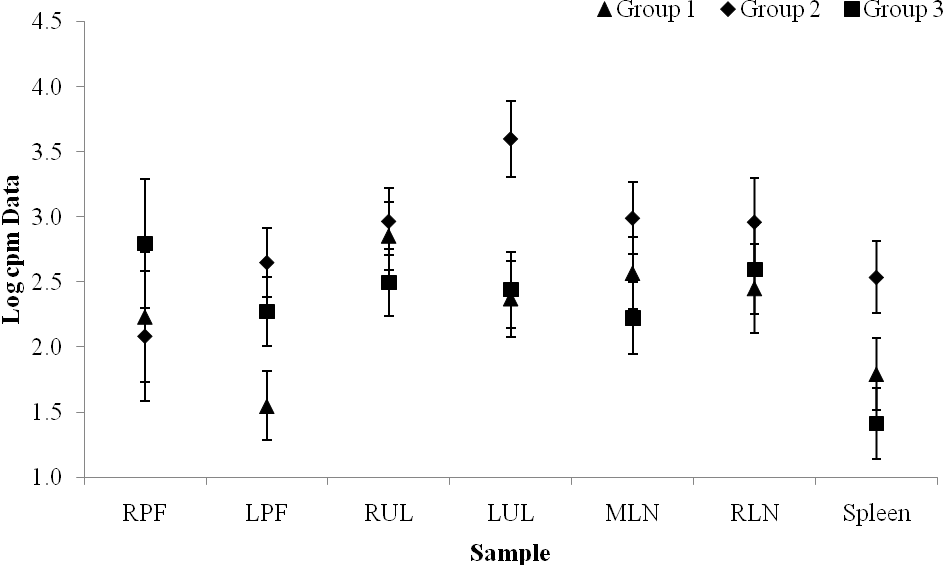


**D**  Day 56 pi


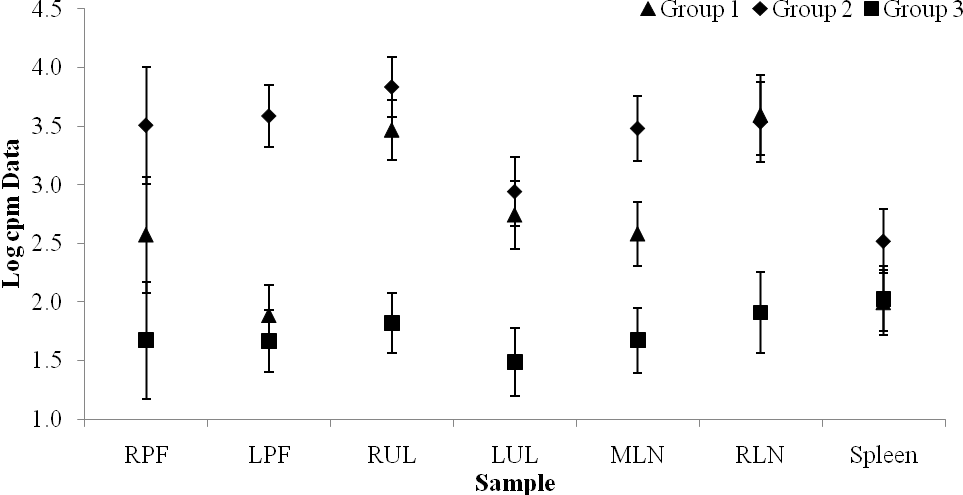


**Proliferative responses of maternal lymph node and spleen samples following stimulation with NCA for 5 days.**

Lymph node and spleen tissue samples were collected at post mortem examination and processed to determine levels of *Neospora*-specific proliferation. The processed cells were stimulated with NCA for 5 days (37 °C in a humidified 5% CO_2_ atmosphere), with 18.5kBq ^3^H Thymidine / well being added for the final 18 h, before being harvested onto glass-fibre filters. The data was then log_10_ transformed before analysis using a linear mixed model. **(A)** day 14 pi **(B)** day 28 pi **(C)** day 42 pi **(D)** day56 pi.

-▲- Group 1 (iv), -♦- Group 2 (sc), -■- Group 3 (Control) Error Bars (±S. E.)
