# Supplementary material for: Maternal and foetal immune responses of cattle following an experimental challenge with Neospora caninum at day 70 of gestation
Source: Vet Res. 2012 Apr 26;43(1):38. doi: 10.1186/1297-9716-43-38 (PMC3416710; doi:10.1186/1297-9716-43-38)
Supplement: Additional file 5 — Levels of antigen specific-IFN-γ (ng/ml) produced by maternal lymph node and spleen samples following stimulation for 4 days with NCA. [file 1297-9716-43-38-S5.doc]

Additional file 5: Levels of antigen specific-IFN-γ (ng/mL) produced by maternal lymph node and spleen samples following stimulation for 4 days with NCA.

**A.**

-▲- Group 1 -♦- Group 2 -■- Group 3


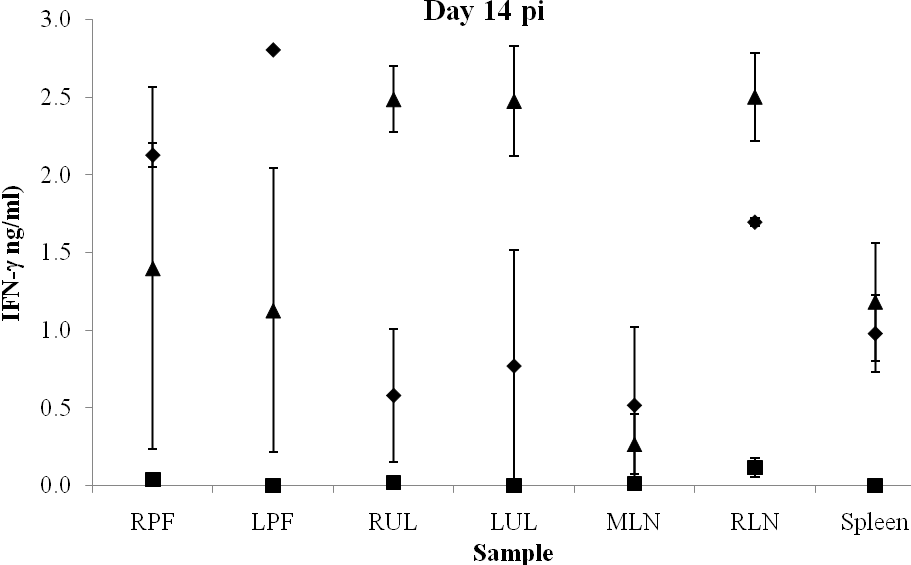


**B.**

-▲- Group 1 -♦- Group 2 -■- Group 3

**
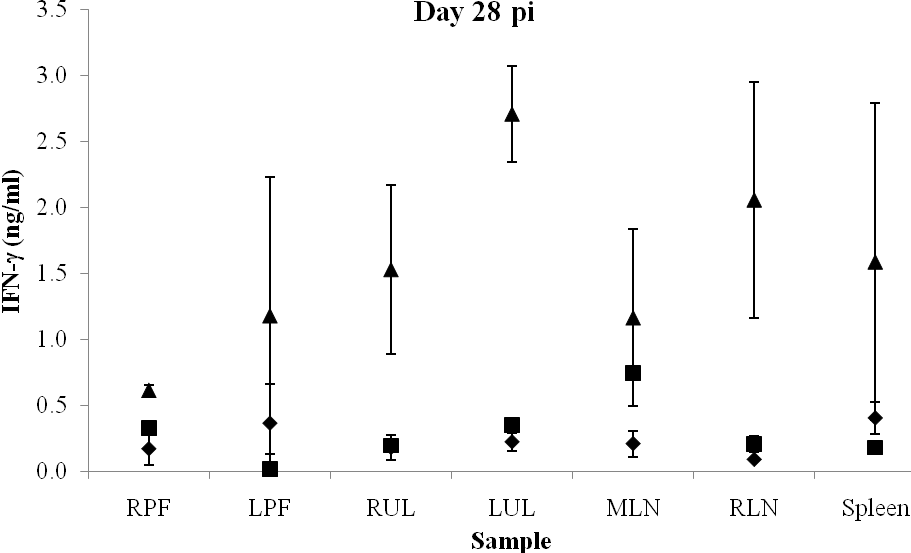
**

C.

-▲- Group 1 -♦- Group 2 -■- Group 3


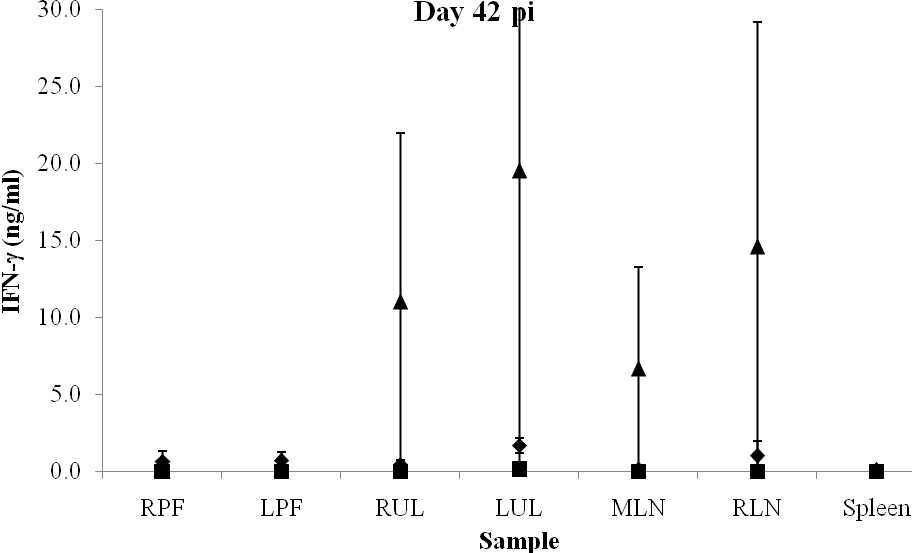


**D.**

-▲- Group 1 -♦- Group 2 -■- Group 3

**
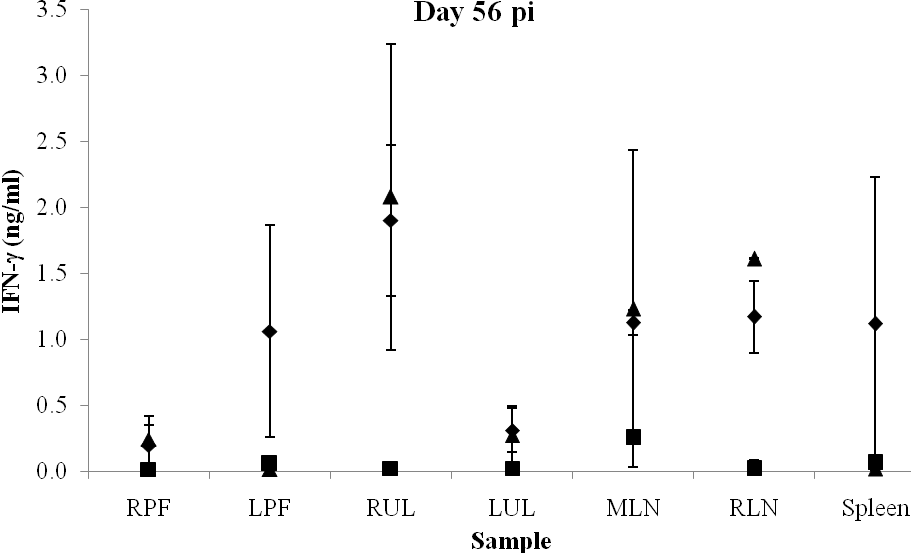
**

**Levels of antigen specific-IFN-γ (ng/mL) produced by maternal lymph node and spleen samples following stimulation for 4 days with NCA.**

Lymph node and spleen tissue samples were collected at post mortem examination and processed to determine levels of *Neospora*-specific proliferation. The processed cells were stimulated with NCA for 4 days (37 °C in a humidified 5% CO_2_ atmosphere); ELISA were performed to determine the concentration of IFN-γ produced. Error bars (S.E.).

-▲- Group 1 -♦- Group 2 -■- Group 3
